# Abalone Viscera Fermented with *Aspergillus oryzae* 001 Prevents Pressure Elevation by Inhibiting Angiotensin Converting Enzyme

**DOI:** 10.3390/nu15040947

**Published:** 2023-02-14

**Authors:** Natsumi Iwamoto, Asahi Sasaki, Tomoaki Maizawa, Naoko Hamada-Sato

**Affiliations:** 1 Course of Safety Management in Food Supply Chain, Tokyo University of Marine Science and Technology, Konan-4, Minato-ku, Tokyo 108-8477, Japan; 2 Department of Ocean Sciences, Tokyo University of Marine Science and Technology, Konan-4, Minato-ku, Tokyo 108-8477, Japan; 3 Research and Development Department, Bull-Dog Sauce Co., Ltd., 3-6-1, Mitsuwa, Kawaguchi-shi, Saitama 334-0011, Japan

**Keywords:** abalone viscera, *Aspergillus oryzae*, hypertension, fermentation, spontaneously hypertensive rats

## Abstract

Abalone viscera, which accounts for more than 20% of the total weight of abalone, is generally regarded as waste in the food industry, and effective methods are required to utilize it productively. In this study, the viscera were fermented with *Aspergillus oryzae* 001 to add functionality. Fermented abalone viscera exhibited increased angiotensin I-converting enzyme (ACE) inhibitory activity and enhanced inhibition of blood pressure elevation in spontaneously hypertensive rats (SHRs). Abalone viscera administration had no significant effect on body weight, food intake, liver and kidney weights, or serum components in SHRs. ACE inhibitors specific to fermented abalone viscera were identified through extraction, fractionation, purification, and analysis. The identified substance was L-*m*-tyrosine, which non-competitively inhibited ACE and, in a single oral administration, significantly reduced blood pressure in SHRs compared to that in the control. This study identified that abalone viscera fermented by *A. oryzae* 001 has an inhibitory effect on blood pressure elevation, suggesting its potential use as a functional food. In addition, L-*m*-tyrosine, a unique substance in fermented abalone viscera, was isolated for the first time as a single ACE-inhibitory amino acid.

## 1. Introduction

The food processing industry produces enormous amounts of organic residues and wastewater, most of which is left unused or untreated [1]. Because waste is detrimental to the environment and human and animal health, various effective ways of using it are being explored [1]. Abalone is one of the most popular and economically important seafood species [2]. Therefore, abalone aquaculture has been increasing worldwide, reaching 190,000 tons per year in 2019 [2,3]. Abalone viscera, which accounts for 15–25% of the total weight, is usually not considered edible and is discarded, contributing to environmental pollution [4,5]. Therefore, effective ways to utilize it are being explored, including the purification of sulfated polysaccharides and antioxidant peptides with bioactive properties from the viscera [6,7]. In addition, silage made from abalone viscera is rich in protein and promotes growth in animals that consume it [8].

*Aspergillus oryzae*, one of the koji molds, has been used for over 2000 years for food fermentation and for over 50 years for the production of food enzymes [9]. Therefore, *A. oryzae* is enlisted in the U.S. Food and Drug Administration’s (FDAs) “Generally Recognized as Safe (GRAS)” list [10]. It produces a variety of enzymes, including proteases and amylases that digest proteins and starch, respectively [11]. The fermented food *idli* increases gamma-aminobutyric acid content, angiotensin I-converting enzyme (ACE) inhibitory activity, and antioxidant activity through koji mold fermentation [12]. The in vivo evaluation of spontaneously hypertensive rats (SHR) with koji mold fermented *idli* has confirmed its blood pressure-lowering effect [12]. Koji mold is also used for effective utilization of waste and the residue generated when walnut oil is extracted (walnut meal) has similarly been reported to enhance ACE inhibitory and antioxidant activities via koji molds fermentation [13].

Hypertension is a major risk factor for cardiovascular disease, including coronary artery disease, left ventricular hypertrophy, valvular heart disease, and arrhythmias, such as atrial fibrillation, stroke, and renal failure [14]. It is estimated that 1.4 billion people worldwide have hypertension, but only 14% have it controlled [15]. Angiotensin-converting enzyme (ACE) catalyzes the conversion of angiotensin I to the vasoconstrictor angiotensin II [16]. Synthetic ACE inhibitors, such as captopril, lisinopril and enalapril are currently used to treat hypertension but have significant side effects including taste abnormalities, rash, cough, hypotension, renal failure, and hyperkalemia [17,18]. By contrast, naturally occurring ACE inhibitors are considered safe [19]. To date, ACE inhibitors have been found in fermented milk [20] and fish surimi [21], rabbit meat [19], and ACE inhibitory peptides have been isolated from those foods and other sources.

With the increase in abalone production, the amount of abalone viscera that is discarded is expected to increase in the future. Therefore, more effective ways to utilize abalone viscera will be required than those at present. In previous studies, abalone viscera fermented with the lactic acid bacteria *Lacticaseibacillus casei* 001 and *Lactiplantibacillus pentosus* SN001 showed ACE-inhibitory activity [22,23]. In addition, single and long-term administration of the fermented products reduced SHR blood pressure and growth inhibition was also not observed. Koji mold, such as lactic acid bacteria, has been traditionally used to ferment foods. In the present study, abalone viscera were fermented with *A. oryzae* 001, and the inhibitory effect of the fermented product on elevated blood pressure was evaluated.

## 2. Materials and Methods

### 2.1. Material and Reagents

Abalone viscera were sourced from Australian farmed blacklip abalone (*Haliotis ruber*) and transported frozen. *A. oryzae* 001 is a proprietary fungus owned by the laboratory. L-tyrosine, soybean oil, isoflurane, tert-butylhydroquinone (TBHQ), L-cysteine, trifluoroacetic acid, sodium hydroxide, *o*-phthalaldehyde solution, phosphorus acid and kits for cholesterol, HDL-cholesterol, and triglyceride E tests as well as glucose and transaminase CII tests, were purchased from Wako Pure Chemical Industries (Osaka, Japan). Potato dextrose broth (PDB) was purchased from Funakoshi Co., Ltd. (Tokyo, Japan). The ACE Kit-WST was purchased from Dojindo Laboratories (Kumamoto, Japan). β-corn starch, casein, α-corn starch, sucrose, AIN76 mineral mixture, AIN76A vitamin mixture without choline deuterium tartrate, and cellulose were purchased from Oriental Yeast Co., Ltd. (Tokyo, Japan). Acetonitrile and distilled water were purchased from KOKUSAN CHEMICAL Co., Ltd. (Tokyo, Japan). ACE from rabbit lung was purchased from Sigma–Aldrich (St. Louis, MO, USA), and D-tyrosine was purchased from NACALAI TESQUE, Inc. (Kyoto, Japan). DL-*o*-tyrosine was purchased from Tokyo Chemical Industry Co., Ltd. (Tokyo, Japan). L-*m*-tyrosine was purchased from Cosmo Bio Co., Ltd. (Tokyo, Japan); D-*m*-tyrosine was purchased from Santa Cruz Biotechnology, Inc. (Dallas, TX, USA), Hip-His-Leu was purchased from Bachem AG (Bubendorf, Switzerland), and His-Leu was purchased from Peptide Institute (Osaka, Japan).

### 2.2. Fermentation by A. oryzae 001

Abalone viscera were lyophilized, ground with a mixer, and sieved through a 500-mesh sieve. The abalone viscera powder was stored at −20 °C until use. *A. oryzae* 001 was activated from −80 °C storage by pre-culturing with shaking (28 °C, 160 rpm for 24 h) in PDB medium. For fermentation, 1 mL of the pre-cultured bacterial solution was added to 100 mL of distilled water with 1 g of abalone viscera powder, and cultured with shaking (28 °C, 160 rpm, 6 d). The culture supernatant of the ferment was subjected to measurement of ACE inhibitory activity. The ACE-inhibitory activity was determined using the ACE Kit-WST according to the manufacturer’s instructions.

### 2.3. Long-Term Administration Study

Eighteen 14-week-old male SHR/Izm rats (Sankyo Lab Service, Tokyo, Japan) were housed in a room at 25 ± 3 °C, with a humidity of 45 ± 5%, and a 12 h light/dark cycle (8:00–20:00 light period). Water (tap water) and feed were provided ad libitum. The rats were pre-reared for 1 week to acclimatize them to the environment. During the pre-rearing period, all rats were fed the same diet. The pre-reared rats were divided into control, fermented, and unfermented groups of 6 rats each and were fed the diets listed in Table 1. The diets for the fermented and unfermented groups contained 5% fermented and unfermented abalone viscera, respectively. Blood pressure was monitored twice a week using a non-observational blood pressure monitor for mice and rats (Blood Pressure Monitor For Mice & Rat Model MK-2000, Muromachi Kikai Co., Tokyo, Japan) six times per animal. Body weight and food intake were also measured on the same day as the blood pressure measurements. Food intake was measured from the difference between the amount fed and the amount remaining. Blood samples were collected under isoflurane anesthesia after one night of fasting, from day 49. After the rats were euthanized, their kidneys and livers were removed for observation and weighing. Blood tests included serum total cholesterol, HDL-cholesterol, glucose, triglyceride, aspartate aminotransferase (AST), and alanine aminotransferase (ALT) activity, as measured using kits (Cholesterol E Test, HDL-cholesterol E Test, Glucose CII Test, Triglyceride E Test, and Transaminase CII Test).

### 2.4. Purification of ACE Inhibitor Components

Fermented and unfermented abalone viscera were extracted with water (50 °C, 125 spm, 60 min) and centrifuged (13,000× *g*, 10 min). The aqueous extract was ultrafiltered using a centrifugal ultrafiltration unit Vivaspin 20 (Sartorius Stedim Biotech GmbH, Göttingen, Germany) with molecular mass cut-off (MWCO) values of 3, 10, 30, and 100 kDa. Each fraction (<3 kDa, 3~10 kDa, 10~30 kDa, 30~100 kDa, >100 kDa) was concentrated in a rotary evaporator, lyophilized, and measured for ACE-inhibitory activity. The fraction with high ACE-inhibitory activity was dissolved in distilled water, filtered through a 0.22 μm filter, and analyzed using reversed-phase high-performance liquid chromatography (RP-HPLC). ODS-120T (4.6 × 250 mm; Tosoh Bioscience, Tokyo, Japan), and liquid A (0.1% trifluoroacetic acid solution) and liquid B (0.1% trifluoroacetic acid solution/acetonitrile = 3:7 mixture) were used as the column and mobile phase, respectively. For elution, a concentration gradient of 0–50% ratio of solution B was applied over 40 min. The flow rate was set at 1.0 mL/min, and the detector at 220 nm. The peak with high ACE-inhibitory activity, unique to aqueous extracts of fermented abalone viscera, was collected and purified repeatedly. The peak with high ACE-inhibitory activity was subjected to a concentration gradient from 7% to 7.7% acetonitrile, and ACE-inhibitory activity was determined according to the manufacturer’s protocol for the ACE Kit-WST, and IC_50_ was calculated.

### 2.5. Identification of ACE Inhibitors

The purified fractions were subjected to Edman degradation, and the purified products were identified using mass spectrometry. Phenylthiohydantoin derivatives produced by Edman degradation were separated and analyzed using RP-HPLC using Zaplous alpha, Pep C18 120A (0.1 × 150 mm; AMR, Inc., Tokyo, Japan). The molecular weights of the purified materials were determined using an LTQ-Orbitrap XL mass spectrometer (Thermo Fisher Scientific K.K., Tokyo, Japan). The ACE-inhibitory activities of various isomers (L-tyrosine, D-tyrosine, DL-*o*-tyrosine, L-*m*-tyrosine, and D-*m*-tyrosine) of the purified substance were determined, and the IC_50_ values were calculated. The structure of the purified product was determined by comparing the IC_50_ of the purified product with those of the various isomers. To eliminate foreign substances in the reagents, standards of all isomers were purified using HPLC and used for the measurement of ACE-inhibitory activity. HPLC conditions were the same as those used for the purification of ACE inhibitory components.

### 2.6. Estimation of Mode of Inhibition

The mode of inhibition was determined using Lineweaver–Burk plots [19,24]. Briefly, 50 μL of L-*m*-tyrosine (0, 0.28, and 0.57 mM) and 100 μL of ACE (10 mU/mL) were mixed and incubated at 37 °C for 10 min. After incubation, 25 μL of Hip-His-Leu (2.5, 5.0, 12.5, and 25 mM) was added, and the mixture was incubated at 37 °C for 40 min. Then, 50 μL of 1N NaOH was added, and after the reaction was stopped, 10 μL of 0.2% *o*-phthalaldehyde solution was added, and the reaction was carried out at room temperature for 15 min under light-shielding conditions. Then, 15 μL of 3.6 M phosphoric acid solution was added, and fluorescence intensity was measured at excitation and emission wavelengths of 360 and 460 nm, respectively. The Michaelis–Menten constant (K_m_) and the maximum reaction rate (V_max_) were calculated according to the Michaelis–Menten kinetic equation from the Lineweaver–Burk plot.

### 2.7. Single-Dose ACE Inhibitor Study

Male SHRs/Izm rats were purchased and housed as described in Section 2.3. Water (tap water) and feed were provided ad libitum, and the rats were pre-reared for at least 1 week to acclimatize to the environment. L-*m*-tyrosine solution (pH 3, 10 mg/kg body weight) or water (pH 3) was orally administered to each rat. The pH was adjusted to 3 because L-*m*-tyrosine is insoluble in water under neutral pH. Blood pressure was measured 6 times per animal before and 2, 4, 6, 8, and 24 h after administration using a non-observational blood pressure monitor.

### 2.8. Statistical Analysis

The blood pressure measurements and weight changes are expressed as mean ± standard error, and other results are expressed as mean ± standard deviation. Rejection was performed using the Smirnov–Grubbs test. Multiple comparisons were performed using the Steel–Dwass test, and comparisons between two test intervals were performed using the t-test. Statistical significance was set at *p* < 0.05.

## 3. Results & Discussion

### 3.1. Long-Term Dosing Study

**Figure 1:**
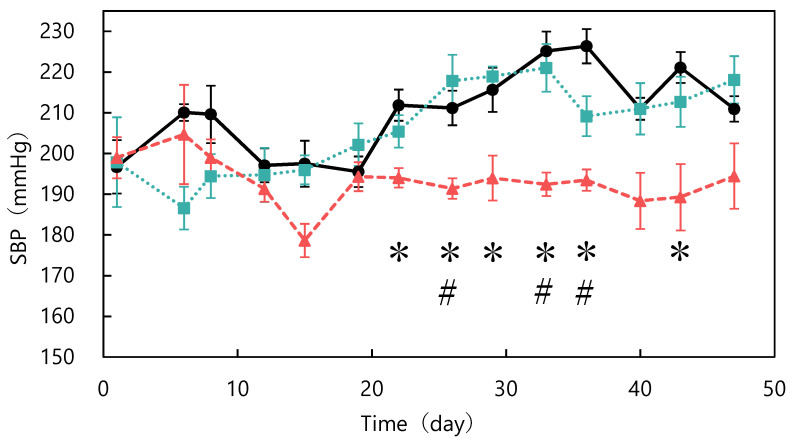
Changes in systolic blood pressure in spontaneously hypertensive rats fed different diets: control (●), unfermented (■), and fermented (▲). * *p* < 0.05 fermented vs. control group; # *p* < 0.05 fermented vs. unfermented group. The data represented the mean values ± standard error (*n* = 6).

**Figure 2:**
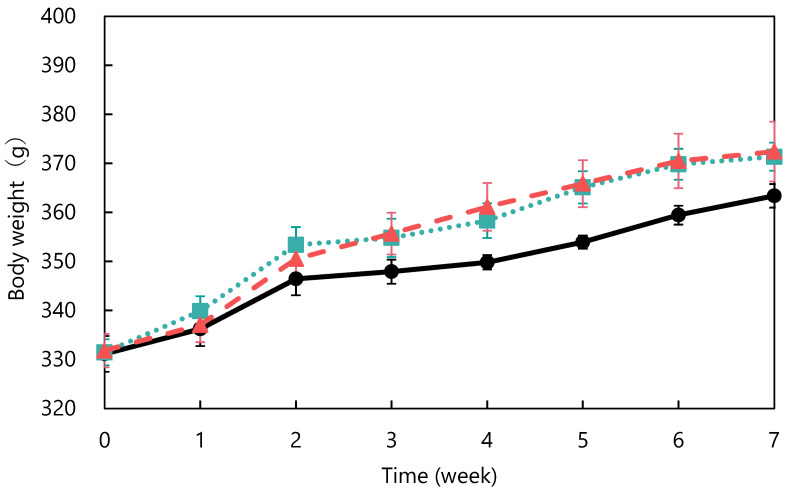
Changes in body weight in spontaneously hypertensive rats: control (●), unfermented (■) and fermented (▲). The data represented the mean values ± standard error (*n* = 6).

The ACE-inhibitory activity of abalone viscera fermented with *A. oryzae* 001 was 56.9%. Fermentation of abalone viscera using lactic acid bacteria requires the addition of glucose [22,23], while the addition of nutrients was not necessary because koji mold has a variety of enzymes. The results of *A. oryzae* 001 fermented and unfermented abalone viscera administered to SHRs are shown in Figure 1. No significant differences in food intake were observed between the test groups during the study period (data not shown). The blood pressure of the fermented group was always lower than that of the control group from day 6 onward. The fermented group always had lower blood pressure than the control and unfermented groups from day 12 onward. Blood pressure was significantly lower in the fermented group than in the control group on days 22, 26, 29, 33, 36, and 43, and significantly different from the unfermented group on days 26, 33, and 36. The results of body weight changes are shown in Figure 2. There were no significant differences in the body weights of SHRs between the test groups during the study period. The average kidney and liver weights are shown in Table 2, and the blood test results are shown in Table 3. The control group was reduced to *n* = 5 due to hemolysis in the serum of one animal in the control group. There were no significant differences in kidney and liver weights between the study groups and no differences in appearance. Serum total cholesterol, HDL-cholesterol, glucose, triglyceride, ALT, and AST levels were also not significantly different between the groups. Rodents are commonly used as animal models of hypertension, with SHRs being the most commonly used model in studies of essential hypertension in humans [25,26,27]. In the present study, the group treated with fermented abalone viscera had consistently lower blood pressure than the other groups after 12 days of treatment. Thus, fermentation with *A. oryzae* 001 imparted an antihypertensive effect to abalone viscera, and the fermented viscera was shown to suppress blood pressure elevation in vivo over the long term. In a previous study, *L. casei* 001 fermented abalone viscera mixed feed suppressed blood pressure elevation of SHRs after 28 days of administration [22], but *A. oryzae* 001 fermented abalone viscera mixed feed suppressed blood-pressure elevation from day 12 of administration. Therefore, it was suggested that in *A. oryzae* 001 fermented abalone viscera suppressed blood pressure elevation more rapidly than *L. casei* 001 fermented abalone viscera in vivo. In a study on *L. pentosus* SN001 fermented abalone viscera, SHRs were reared for 9 weeks and *L. pentosus* SN001 fermented abalone viscera mixed feed suppressed blood pressure elevation from week 8 of rearing [23], and no significant differences occurred between the fermented and unfermented groups [23]. The fermented group showed significantly lower blood pressure than the unfermented group during approximately 7 weeks three times (on days 26, 33, and 36) in this experiment. Therefore, *A. oryzae* 001 fermentation may have greatly enhanced the inhibition of blood pressure elevation in abalone viscera compared to *L. pentosus* SN001 fermentation. These results suggest that *A. oryzae* 001 fermentation enhanced the inhibition of blood pressure elevation in abalone viscera, and that the in vivo effect was stronger than that of lactic-acid fermentation. Significant differences in food intake and body weight between test groups have been used as an indicator of growth inhibition in rats [23,28]. Administration of the abalone viscera mixture did not affect food intake or body weight, suggesting that *A. oryzae* 001 fermented and unfermented abalone viscera did not inhibit SHR growth. Hypertensive patients tend to have lower HDL cholesterol levels and higher triglyceride levels, and total cholesterol levels above a certain level induce a greater increase in blood pressure [29]. Triglyceride and total cholesterol levels in the unfermented group tended to be higher than in the control group. The fermented group tended to have lower total cholesterol than the unfermented group. Abalone viscera contains about 10% lipids in its dried state, and a diet high in lipids increases cholesterol and triglyceride levels [23,30]. *A. oryzae* has lipolytic enzymes and has used fatty acids as a carbon source in previous reports [31]. Therefore, *A. oryzae* 001 may be degraded and reduced lipids in abalone viscera, resulting in lower total cholesterol levels in the fermented group than in the unfermented group. Hyperglycemia occurs due to abnormalities in glucose regulation, such as decreased glucose utilization, increased glucose production, and insulin secretion [32]. Since there were no significant differences in glucose concentrations among the study groups, it appears that abalone viscera consumption does not induce hyperglycemia. The reason for the highest glucose concentration in the fermented group may be due to the high carbohydrate-degrading enzyme activity of the fermented abalone viscera, since *A. oryzae* produces α-amylase and glucoamylase [11]. ALT and AST activities are indicators of liver health [33]. These values and liver appearance and weight displayed no significant differences between the test groups and suggested that abalone viscera consumption does not affect the liver. Long-term administration of feed mixed with *idli* fermented with *A. oryzae* suppressed the increase in blood pressure in SHRs from at least day 14, and ALT and AST activities remained normal with no significant difference from control or unfermented for 10 weeks [12]. Thus, it was suggested that fermentation with *A. oryzae* did not affect ALT and AST activities.

### 3.2. Purification of ACE inhibitors

**Figure 3:**
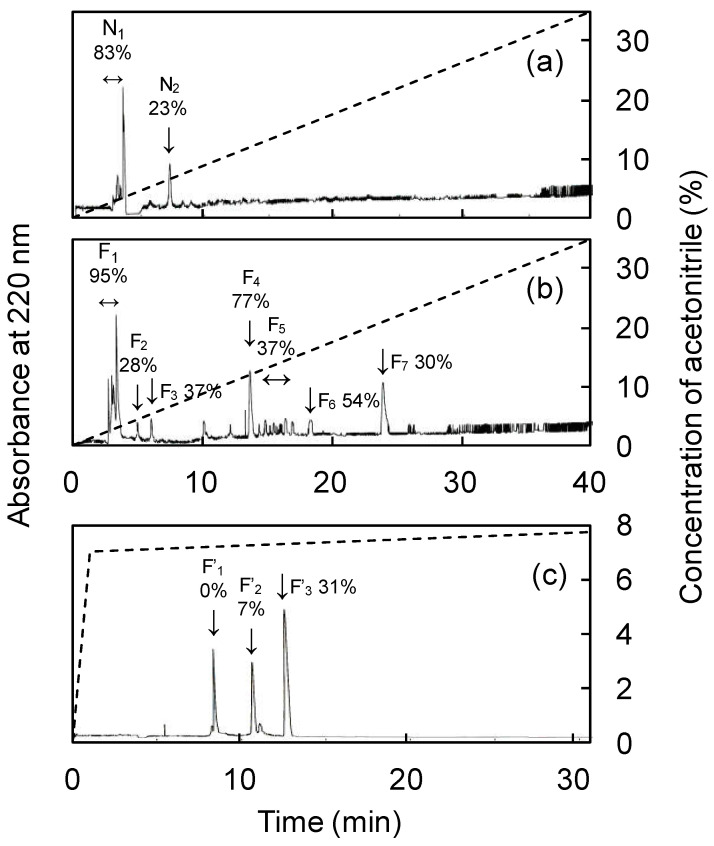
Separation of <3 kDa fraction of fermented and unfermented abalone viscera by RP-HPLC. (**a**) Chromatogram of unfermented abalone viscera, (**b**) chromatogram of fermented abalone viscera, (**c**) chromatogram of fraction F_4_. The percentage of ACE inhibition for each peak is indicated next to the symbol.

The IC_50_ values of each fraction separated by ultrafiltration are listed in Table 4. The fermentation products showed maximum ACE-inhibitory activity and a weight of <3 kDa. Therefore, the <3 kDa fraction was further analyzed. The results of RP-HPLC analysis of the <3 kDa of fermented and unfermented products are shown in Figure 3. The peaks of the fermented and unfermented products were designated as (F_1_–F_7_) and (N_1_–N_2_), respectively, with larger peaks detected in F_1_ and F_4_. F_1_ was similar in retention time and size to N_1_, so F_4_ was considered to be the fermentation product-specific peak; F_4_ was further purified using RP-HPLC to yield three peaks (F’_1_–F’_3_). The ACE-inhibitory activity was not observed in F’_1_ and F’_2_, but high ACE-inhibitory activity was observed in F’_3_ and it was subjected to Edman degradation and mass spectrometry. Protease activity involved in protein degradation was found to be enhanced during fermentation (Data not shown). Abalone viscera is rich in protein and fermentation is used as an effective means of protein hydrolysis [34]. Fermented camel and bovine milk showed maximum ACE-inhibitory activity in the <3 kDa and <5 kDa fractions, respectively [35,36]. Fermented soybean showed strong ACE-inhibitory activity in the lower molecular weight fraction, with maximum activity in the <2 kDa fraction [37]. Those ferments showed higher ACE-inhibitory activity in the smaller molecular weight fractions, consistent with the results of the present study. Previous studies have confirmed that the smaller the molecular weight of a bioactive substance, the easier it passes through the intestinal wall and the more likely it is to exert its effect in vivo [38,39]. Thus, *A. oryzae* 001 fermented abalone viscera had high ACE-inhibitory activity in the low molecular weight fraction, suggesting that it is effective in vivo.

### 3.3. Identification of ACE Inhibitors

Edman degradation analysis showed that only one tyrosine residue was present in the purified substance. Mass spectrometry results showed an *m*/*z* of 182.08122 and a composition of C_9_H_12_O_3_N. Thus, it was clear that the purified product was tyrosine. Since tyrosine has many isomers, the structure was determined by measuring the ACE-inhibitory activity of the various isomers and comparing the IC_50_ with that of the purified product. The IC_50_ values for each isomer were as follows: L-tyrosine and D-tyrosine showed less than 50% ACE inhibition at all concentrations. The IC_50_ values for the ACE inhibition of DL-*o*-tyrosine, L-*m*-tyrosine, and D-*m*-tyrosine were 0.62 mg/mL, 0.31 mg/mL, and 0.96 mg/mL, respectively. L-*m*-tyrosine was the most potent ACE inhibitor, with an IC_50_ value comparable to that of the isolated peak. Therefore, F’_3_ was determined to be L-*m*-tyrosine. Tyrosine is effective for mental health, and dietary tyrosine intake has been found to improve cognitive performance and physical performance tasks that are sensitive to it [40]. ACE inhibitors of natural origin were present in carp scales, salmon processing by-products, and aosa-derived substances [41,42,43]. Previous studies have reported tyrosine-containing dipeptides [39,44,45] and tripeptides over [46,47] ACE inhibitory peptides. Inhibitory dipeptides with a tyrosine residue at the C-terminus are effective [48,49]. Tyrosine-containing peptides may also be effective because of the high ACE-inhibitory activity of tyrosine. Since the amount of tyrosine contained in abalone viscera is not high [50], it is assumed that it was purified by fermentation.

### 3.4. Estimation of Mode of Inhibition

**Figure 4:**
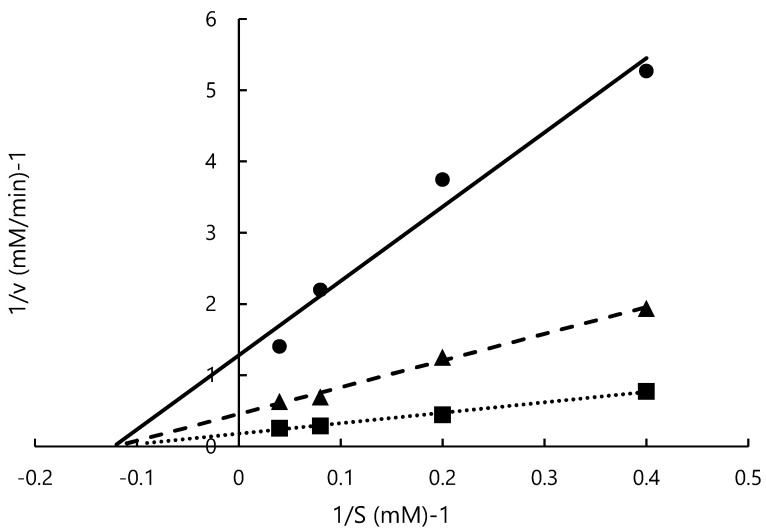
Lineweaver–Burk plot of L-*m*-tyrosine. 0 mM (■), 0.28 mM (▲), 0.57 mM (●).

In previous studies, ACE-inhibitory activity and the mode of inhibition of peptides containing tyrosine were measured [44,51], but the mode of inhibition of tyrosine alone or L-*m*-tyrosine was not determined. This study is the first to investigate the ACE-inhibitory activity of L-*m*-tyrosine. Lineweaver–Burk plots of ACE activity at various concentrations of L-*m*-tyrosine (0, 0.28, 0.57 mM) are shown in Figure 4. V_max_ was 5.56, 2.20, and 0.78 mM/min, respectively, and was concentration dependent. K_m_ was similar at 8.15, 8.24, and 8.14 mM, respectively. From the slope and y-axis intercept, K_i_ was 0.12 mM. V_max_ was concentration-dependent while K_m_ was relatively constant suggesting that ACE inhibition by L-*m*-tyrosine is a non-competitive inhibition. Similar to the present study, several tyrosine-containing dipeptides noncompetitively inhibited ACE [44]. In previous reports, ACE inhibitors obtained by hydrolysis of marine products, such as squid and tuna noncompetitively inhibited ACE [52,53]. However, the ACE inhibitory sites of these substances were not identified [52,53]. In most cases, binding of the inhibitor to the allosteric site of the enzyme results in a pattern of noncompetitive inhibition, but there are exceptions. Because ACE inhibitors from different foods are not identical, detailed inhibition methods require further investigation [54].

### 3.5. Single-Dose Study of ACE Inhibitors in SHRs

**Figure 5:**
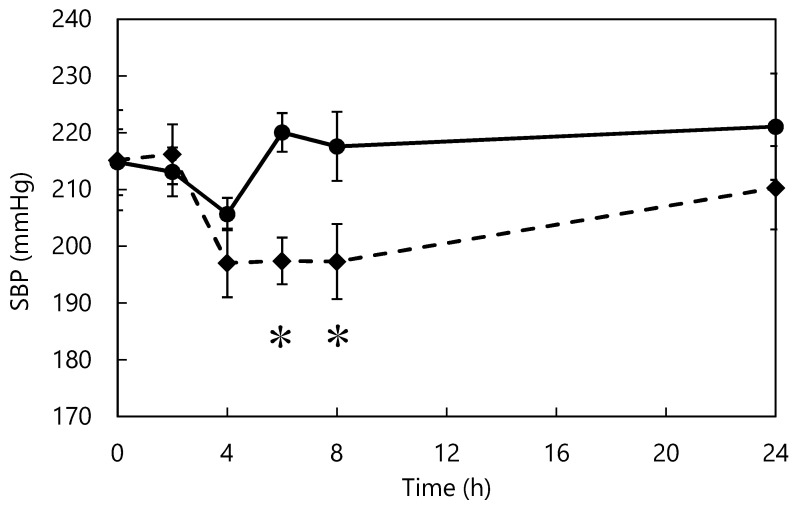
Changes in blood pressure after L-*m*-tyrosine administration. Control (●) and Tyrosine (♦) groups. * *p* < 0.05 fermented vs. control group. The data represent mean ± standard error (*n* = 7).

The effect of L-*m*-tyrosine administration on the blood pressure of SHRs is shown in Figure 5. From 4 h after administration, the tyrosine group showed lower blood pressure than the control group. Six and eight hours after administration, the blood pressure of the tyrosine group was significantly lower than that of the control group. In vivo studies indicate that L-tyrosine-supplemented diets prevent blood pressure elevation and tyrosine-containing peptides reduce blood pressure in SHRs in the short term [55,56]. However, there are no reported studies of L-*m*-tyrosine. L-*m*-tyrosine was identified as the active component that acted as the ACE inhibitor in this study. Tyrosine isomers differ in structure, resulting in differences in ACE-inhibitory activity, behavior in the body, and digestibility [57]. The smaller the molecular weight of a bioactive substance, the faster it is digested and absorbed, and the more rapidly it exerts its effects in vivo [38,39]; therefore, among the isomers of L-tyrosine, L-*m*-tyrosine may be the most potent inhibitor of elevated blood pressure in vivo.

## 4. Conclusions

In this study, abalone viscera, an underutilized resource, was fermented with *A. oryzae* 001, its ACE-inhibitory activity was enhanced, and its inhibition of blood pressure elevation in vivo was confirmed. The ACE inhibitor unique to fermented abalone viscera was identified as L-*m*-tyrosine, which was found to inhibit ACE in a non-competitive manner. Furthermore, L-*m*-tyrosine showed antihypertensive effects in vivo. These results revealed that the fermentation of abalone viscera by *A. oryzae* 001 enhanced the antihypertensive effect of abalone viscera, suggesting that fermented abalone viscera can be utilized as a functional material to inhibit elevated blood pressure. In addition, L-*m*-tyrosine was found, for the first time, to be an amino acid with high ACE-inhibitory activity.

## Figures and Tables

**Table 1:** Diet composition.

Total Ingredients (g/kg)	Control	Unfermented	Fermented
β-Cornstarch	392	345.5	345.5
Milk casein	195	195	195
α-Cornstarch	132	132	132
Sucrose	100	100	100
Soybean oil	70	70	70
Cellulose	50	50	50
AIN-76 Mineral mixture	35	35	35
AIN-76A Vitamin mixture	12.5	12.5	12.5
L-Cysteine	3	3	3
TBHQ	0.014	0.014	0.014
NaCl	10	6.5	6.5
Abalone viscera	0	50	50

**Table 2:** Kidney and liver weights of SHRs in each test section.

Test Group	Control	Unfermented	Fermented
Kidney (g)	2.56 ± 0.13	2.55 ± 0.14	2.47 ± 0.08
Liver (g)	10.06 ± 0.47	10.34 ± 0.43	10.26 ± 0.38

The data represent mean ± standard deviation, *n* = 6.

**Table 3:** Concentration and activity of SHRs components in serum.

Test Group	Control	Unfermented	Fermented
Glucose (mg/dL)	119.0 ± 14.4	114.7 ± 32.7	133.6 ± 23.1
Triglycerides (mg/dL)	69.1 ± 16.2	78.9 ± 13.8	74.0 ± 14.4
Total cholesterol (mg/dL)	76.0 ± 14.5	84.0 ± 11.3	74.4 ± 8.8
HDL-cholesterol (mg/dL)	43.6 ± 2.9	42.1 ± 7.6	43.6 ± 7.2
Alanine aminotransferase activity (IU/L)	25.3 ± 2.4	24.6 ± 1.2	24.3 ± 2.2
Aspartate aminotransferase activity (IU/L)	33.0 ± 7.4	34.4 ± 7.1	31.1 ± 5.9

The data represent mean ± standard deviation, *n* = 5 (Control), *n* = 6 (Unfermented and Fermented).

**Table 4:** Weight of each fraction and IC_50_ of ACE activity.

MWCO (kDa)		<3	3~10	10~30	30~100	100<
Fermented	IC_50_ (mg/mL)	0.36	0.41	0.58	0.42	0.38
	Fraction weight (mg)	451	42	18	10	24
Unfermented	IC_50_ (mg/mL)	0.68	0.49	0.85	N.D.	1.35
	Fraction weight (mg)	230	14	35	N.D.	36

N.D. = not detected.

## Data Availability

The data presented in this study are available on request from the corresponding author.
